# Dosimetric effect of rotational setup errors in stereotactic radiosurgery with HyperArc for single and multiple brain metastases

**DOI:** 10.1002/acm2.12716

**Published:** 2019-09-11

**Authors:** Tomohiro Sagawa, Shingo Ohira, Yoshihiro Ueda, Yuichi Akino, Hirokazu Mizuno, Masao Matsumoto, Masayoshi Miyazaki, Masahiko Koizumi, Teruki Teshima

**Affiliations:** ^1^ Department of Radiation Oncology Osaka International Cancer Institute Osaka Japan; ^2^ Department of Medical Physics and Engineering Graduate School of Medicine Osaka University Suita Japan; ^3^ Division of Medical Physics, Oncology Center Osaka University Hospital Suita Japan

**Keywords:** HyperArc, multiple brain metastases, radiosurgery, rotational setup error

## Abstract

**Purpose:**

In stereotactic radiosurgery (SRS) with single‐isocentric treatments for brain metastases, rotational setup errors may cause considerable dosimetric effects. We assessed the dosimetric effects on HyperArc plans for single and multiple metastases.

**Methods:**

For 29 patients (1–8 brain metastases), HyperArc plans with a prescription dose of 20–24 Gy for a dose that covers 95% (D_95%_) of the planning target volume (PTV) were retrospectively generated (Ref‐plan). Subsequently, the computed tomography (CT) used for the Ref‐plan and cone‐beam CT acquired during treatments (Rot‐CT) were registered. The HyperArc plans involving rotational setup errors (Rot‐plan) were generated by re‐calculating doses based on the Rot‐CT. The dosimetric parameters between the two plans were compared.

**Results:**

The dosimetric parameters [D_99%_, D_95%_, D_1%_, homogeneity index, and conformity index (CI)] for the single‐metastasis cases were comparable (*P *> 0.05), whereas the D_95%_ for each PTV of the Rot‐plan decreased 10.8% on average, and the CI of the Rot‐plan was also significantly lower than that of the Ref‐plan (Ref‐plan vs Rot‐plan, 0.93 ± 0.02 vs 0.75 ± 0.14, *P* < 0.01) for the multiple‐metastases cases. In addition, for the multiple‐metastases cases, the Rot‐plan resulted in significantly higher V_10Gy_ (*P* = 0.01), V_12Gy_ (*P* = 0.02), V_14Gy_ (*P* = 0.02), and V_16Gy_ (*P* < 0.01) than those in the Ref‐plan.

**Conclusion:**

The rotational setup errors for multiple brain metastases cases caused non‐negligible underdosage for PTV and significant increases of V_10Gy_ to V_16Gy_ in SRS with HyperArc.

## 1 INTRODUCTION

Brain metastases are one of the most frequent neurological complications of systemic cancer.^1^ It has been estimated that 20%–40% of cancer patients will develop brain metastases during their disease.^2^ For brain metastasis, various treatment options are available, such as surgical resection, whole‐brain radiotherapy (WBRT), stereotactic radiosurgery (SRS), and dexamethasone supportive therapy.^3^ The optimal option should be chosen after considering patient factors (such as age and performance status), tumor factors (such as extracranial cancer activity, number, size, and location), and outcomes (such as survival, tumor control, and quality of life).^3^ Regarding radiotherapy, WBRT has been the mainstay for brain metastases treatment, but WBRT causes local damage or necrosis of normal tissue within 1 yr with 100% probability and deterioration of cognitive function resulting in poor quality of life.^4^ In contrast, SRS can expose normal tissues to less radiation, preserve neurocognitive function, and minimize radiation‐associated hair loss.^3^, ^4^, ^5^, ^6^, ^7^, ^8^, ^9^ Therefore, SRS has received increased attention as a treatment option for brain metastases.

Lately, advances in technology permit linear accelerator (LINAC)‐based SRS. Especially volumetric‐modulated arc therapy (VMAT) technique provides faster, safer, and more accurate treatment than conventional treatment.^10^ Liu et al. demonstrated that modern LINACs could simultaneously deliver shaped doses to multiple targets and achieve accuracy and precision as high as those of the Gamma Knife^®^ because of the availability of image‐guided radiation therapy, advances in computers, and improvement in tools, such as high‐definition multileaf collimators (MLC).^11^ A new commercially available SRS treatment approach, named HyperArc™, was recently released. This approach is based on the seminal work of a group from the University of Alabama^12^, ^13^ and automatically sets the location of the single‐isocenter, noncoplanar beam arrangement and collimator angle. The HyperArc can provide a steeper dose gradient for targets while minimizing doses to surrounding normal tissues as much as possible with a lesser workload than that of the conventional SRS technique of VMAT.^12^ In addition, single‐isocentric irradiation for multiple metastases can reduce treatment time compared with conventional multi‐isocentric irradiation treatment devices, such as the Gamma Knife and CyberKnife^®^.^12^, ^13^, ^14^, ^15^, ^16^, ^17^, ^18^, ^19^, ^20^

In SRS, setup errors are important considerations.^18^, ^21^ Especially, single‐isocentric SRS for multiple targets is not robust regarding rotational setup errors.^18^ According to a report by Guckenberger et al., the rotational setup error around the three axes in each of 98 patients who had undergone LINAC‐based SRS was ≤1.7°± 0.8° on average, with a 4.0° maximum.^21^ Roper et al. simulated the effect of rotational setup errors for single‐isocentric VMAT‐based SRS for multitargets and showed that the errors could compromise target coverage, especially for small targets far from the isocenter.^18^ The single‐isocentric irradiation using HyperArc plans with a steep dose gradient may be considerably affected by rotational setup errors, and the dose coverage of targets far from the isocenter can be worse.

The aim of this study was to determine the dosimetric effects of rotational setup errors in stereotactic radiosurgery with HyperArc for brain metastases in a clinical setting. A retrospective analysis of 29 patients was performed by comparing two plans: one without rotational setup errors and one with rotational setup errors.

## 2 METHODS

### 2.1 Patients and clinical treatment

This study included 29 patients with 1–8 brain metastases who had undergone single‐fraction SRS for brain tumors between April 2017 and March 2018 at the Osaka International Cancer Institute. The study was approved by our ethics committee, with written informed consent provided by the patients. Thirteen of the patients presented with one metastasis, nine with two metastases, five with three, one with seven, and one with eight. For each patient, a planning computed tomography (pCT) scan was acquired by using a dual‐energy CT scanner (Revolution HD; GE Medical Systems, Milwaukee, WI) with a thermoplastic mask immobilized by using a double‐shell positioning system. The acquisition parameters were a pixel count of 512 × 512, a slice thickness of 1 mm, tube voltages of 80 and 140 kVp, and a field of view of 350 mm. From the acquired CT scan data, a virtual monochromatic image at 77 keV, which provides Hounsfield unit values equivalent to those of a conventional CT scan (120 kVp),^22^ was reconstructed and used for treatment plans.

The gross tumor volume (GTV) was delineated on the CT image by referring to gadolinium‐enhanced T1‐weighted magnetic resonance imaging sets and using a treatment planning system (TPS) Eclipse (version 13.7; Varian Medical Systems, Palo Alto, CA). A clinical target volume (CTV) with a 2‐mm margin was generated from the GTV, and a planning target volume (PTV) was generated by adding an isotropic margin of 1 mm to the CTV. For multiple‐metastases cases, a structure, named PTV_all_, was defined as the union of each PTV in a patient. The prescription dose was 20–24 Gy for a 95% volume of the PTV for the single‐metastasis cases or PTV_all_ for multiple‐metastases cases in a single fraction. Before each treatment, a kilo‐voltage cone‐beam CT (CBCT) scan was acquired, and bone‐matching corrections were performed between the pCT and the CBCT.

### 2.2 HyperArc plans

The pCT sets and structure sets used for original treatment were retrospectively imported to the prototype TPS Eclipse (version 15.5) with beam data from the TrueBeam STx (Varian Medical Systems), which equips a 2.5‐mm leaf‐width MLC. HyperArc plans based on these sets were generated for each patient. The isocenter position is automatically set based on the selected target structures. These structures were used for collimator angle optimization. Arc geometry (four arc fields; one full coplanar arc with a 0° couch and three half noncoplanar arc fields a 315°, 45°, and 90° or 270° couch) arranged with a single isocenter automatically located on the basis of the distance between each lesion.^20^, ^23^ The prescription dose was the same as that of the clinical treatment plan for 95% volume of the PTV_all_, and 6‐MV flattening filter‐free photon beams were used. In the optimization process, the minimum dose in the target structures was set at the prescription dose. An analytical anisotropic algorithm was used in dose calculations of all plans with a 1.25‐mm grid size. The HyperArc plan generated by this process was designated as the reference plan (Ref‐plan).

### 2.3 HyperArc with rotational setup errors

**Figure 1:**
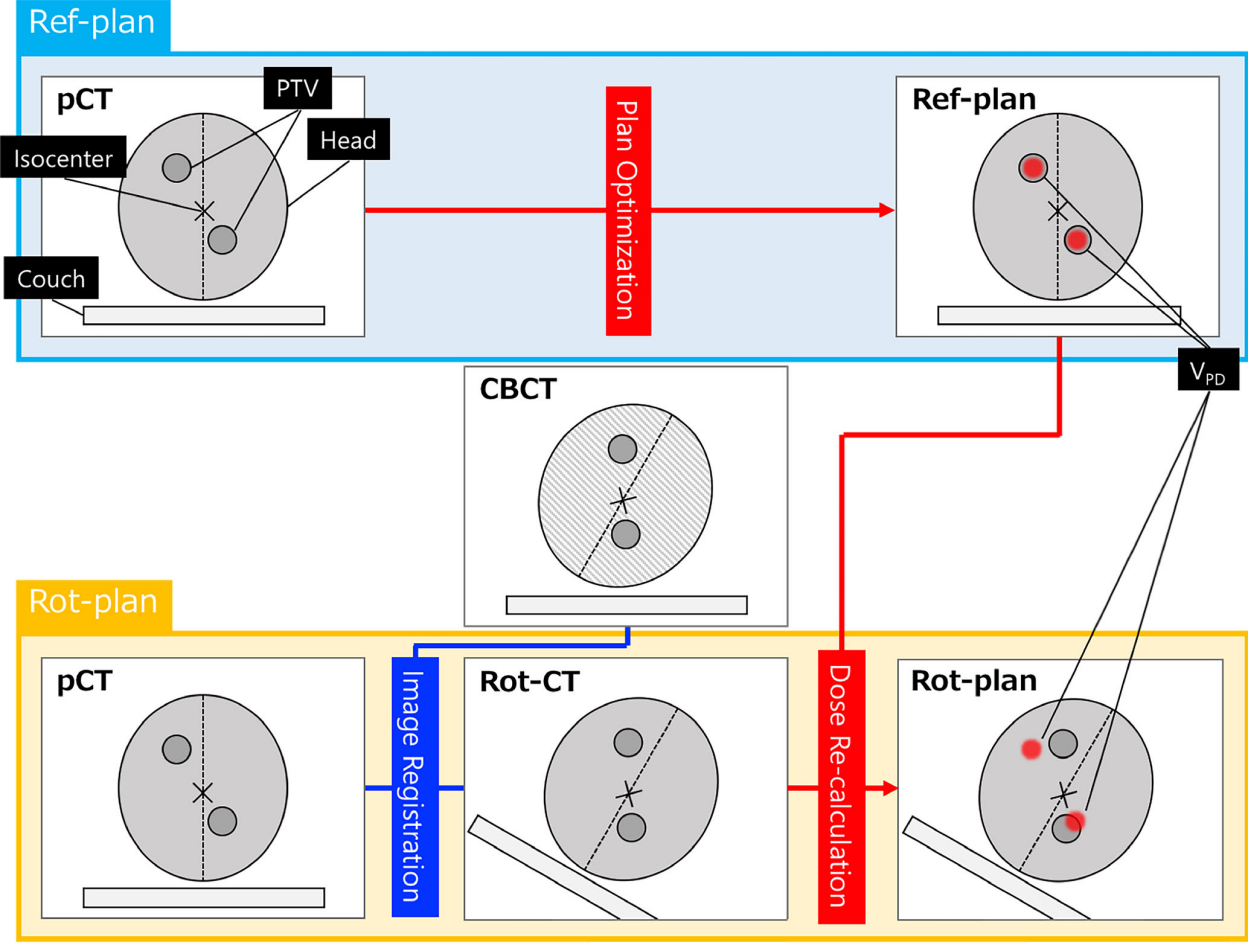
Workflow of how to generate a rotation plan (Rot‐plan). The computed tomography (CT) used for the reference plan (Ref‐plan) and the cone‐beam CT (CBCT) acquired during treatments are registered (Rot‐CT). The Rot‐plans involving rotational setup errors are generated by re‐calculating doses based on the Rot‐CT. The positioning in CBCT includes rotational setup errors. V_PD_ is the prescription isodose volume.

As done against the Ref‐plan, we generated the HyperArc plan with rotational setup errors (Rot‐plan) for each patient (Fig. 1). The pCT set and CBCT set were imported to MIM Maestro (version 6.7; MIM software Inc., USA). The pCT set was rotated in accordance with the rotational setup errors in the CBCT set (Rot‐CT), and all structures in the Ref‐plan were copied to the Rot‐CT. Subsequently, doses were re‐calculated based on the Rot‐CT with the same dose calculation parameters (such as MU, MLC patterns, and Arc geometry) used in the Ref‐plan.

The distance between the position of the isocenter (*x_i_*, *y_i_*, *z_i_*) and that of each target (*x_t_*, *y_t_*, *z_t_*) (ITD) was calculated according to the following formula.

(1) $$\mathrm{ITD}=\sqrt{{\left(x_{t}-x_{i}\right)}^{2}+{\left(y_{t}-y_{i}\right)}^{2}+{\left(z_{t}-z_{i}\right)}^{2}}$$

Further, a 3‐dimensional rotation setup error (RSE_3D_) was defined by [Eq. (2)],

(2) $${\mathrm{RSE}}_{3D}=\sqrt{{\mathrm{RSE}}_{p}^{2}+{\mathrm{RSE}}_{y}^{2}+{\mathrm{RSE}}_{r}^{2}}$$

where RSE*_p_*, RSE*_y_*, and RSE*_r_* were the rotational setup errors for pitch, yaw, and roll axis, respectively.

### 2.4 Data analysis

In this study, we compared the dosimetric parameters between the Ref‐plan and Rot‐plan for targets regarding the D_99%_, D_95%_, D_1%_, the homogeneity index (HI) and conformity index (CI). D_95%_ was the dose that covers 95% of the PTV. The D_99%_ and D_1%_ were defined in the same way. The HI was defined according to Eq. (3):

(3) $$\mathrm{HI}=D_{\max}/D_{\mathrm{prescribed}}$$

where *D*_max_ and *D*_prescribed_ were the maximum and prescribed doses, respectively.^24^

The CI was defined by Paddick as follows,

(4) $$\mathrm{CI}=({\mathrm{PTV}}_{\mathrm{PD}}/\mathrm{PTV})\times \left({\mathrm{PTV}}_{\mathrm{PD}}/V_{\mathrm{PD}}\right)$$

where PTV_PD_ was the volume of PTV covered by the prescription dose, and V_PD_ was the prescription isodose volume.^25^ The relative dose error (RDE) between the Ref‐plan and Rot‐plan was calculated as follows:

(5) $$\mathrm{RDE}=D_{\mathrm{Rot}}-D_{\mathrm{Ref}}/D_{\mathrm{Ref}}\times 100\left[\%\right]$$

where *D*_Ref_ and *D*_Rot_ were the specific doses of the Ref‐plan and Rot‐plan respectively. For organs at risk (OAR), the maximum dose (*D*_max_) for the brainstem and the volume of the OAR receiving at least the dose in the range from 2 Gy to 16 Gy (V_2Gy_–V_16Gy_) for brain tissues, excluding PTVs, of both plans were compared.

We used SPSS (version 24; IBM, USA) for all statistical analyses in this study. Spearman's rank correlation coefficient was performed for derivation of correlation factors. In statistical comparisons between the Ref‐plan and Rot‐plan, a paired Wilcoxon’ signed‐rank test was used. In the case of *P*‐values < 0.05, we rejected the null hypothesis that there was no difference between the Ref‐plan and Rot‐plan. In addition, subgroup analysis (single or multiple metastases) was performed.

## 3 RESULTS

**Figure 2:**
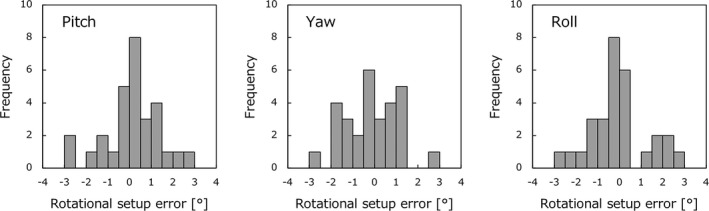
Histograms of rotational setup errors for three axes (Pitch, Yaw, and Roll) for 29 patients.

Figure 2 shows the histogram relative to the rotational setup errors for three axes (pitch, yaw, and roll) for all patients. The means of the relative rotational setup errors were 0.29° ± 1.23°, 0.00° ± 1.22°, and 0.32° ± 1.27° for pitch, yaw, and roll respectively. There were no significant differences in these errors between the three axes (*P *> 0.05). The maximum errors were 3.00° for pitch, 3.00° for yaw, and 2.90° for roll.

**Figure 3:**
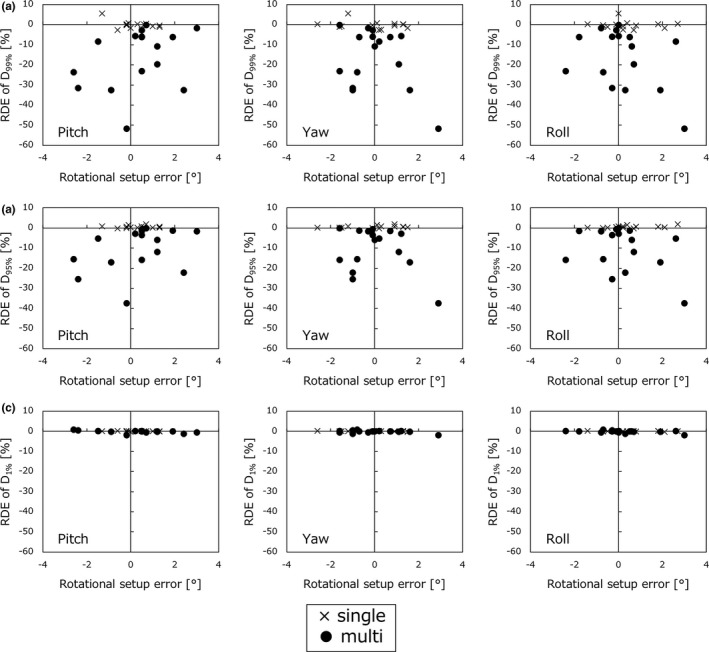
Relative dose error (RDE) of dosimetric parameters for all planning target volumes (PTV_all_), (a) D_99%_, (b) D_95%_, and (c) D_1%_, for single‐ and multiple‐metastases cases by placing rotational setup errors for three axes (Pitch, Yaw, and Roll) on the horizon axis.

Figure 3 illustrates RDE of (a) D_99%_, (b) D_95%_, and (c) D_1%_ for PTV_all_ at three rotation axes. All dose errors for single‐metastasis cases (*n* = 13) were < 2%. The isocenter was set in the same location as a target, so regardless of RSE_3D_, a dosimetric effect mostly could not be detected. In contrast, for the multiple‐metastases cases (*n* = 16), the dose to PTV_all_ decreased by 10.4% ± 10.6% under the influence of setup errors. The maximum differences were −37.4% for the D_95%_ of PTV_all_.

**Figure 4:**
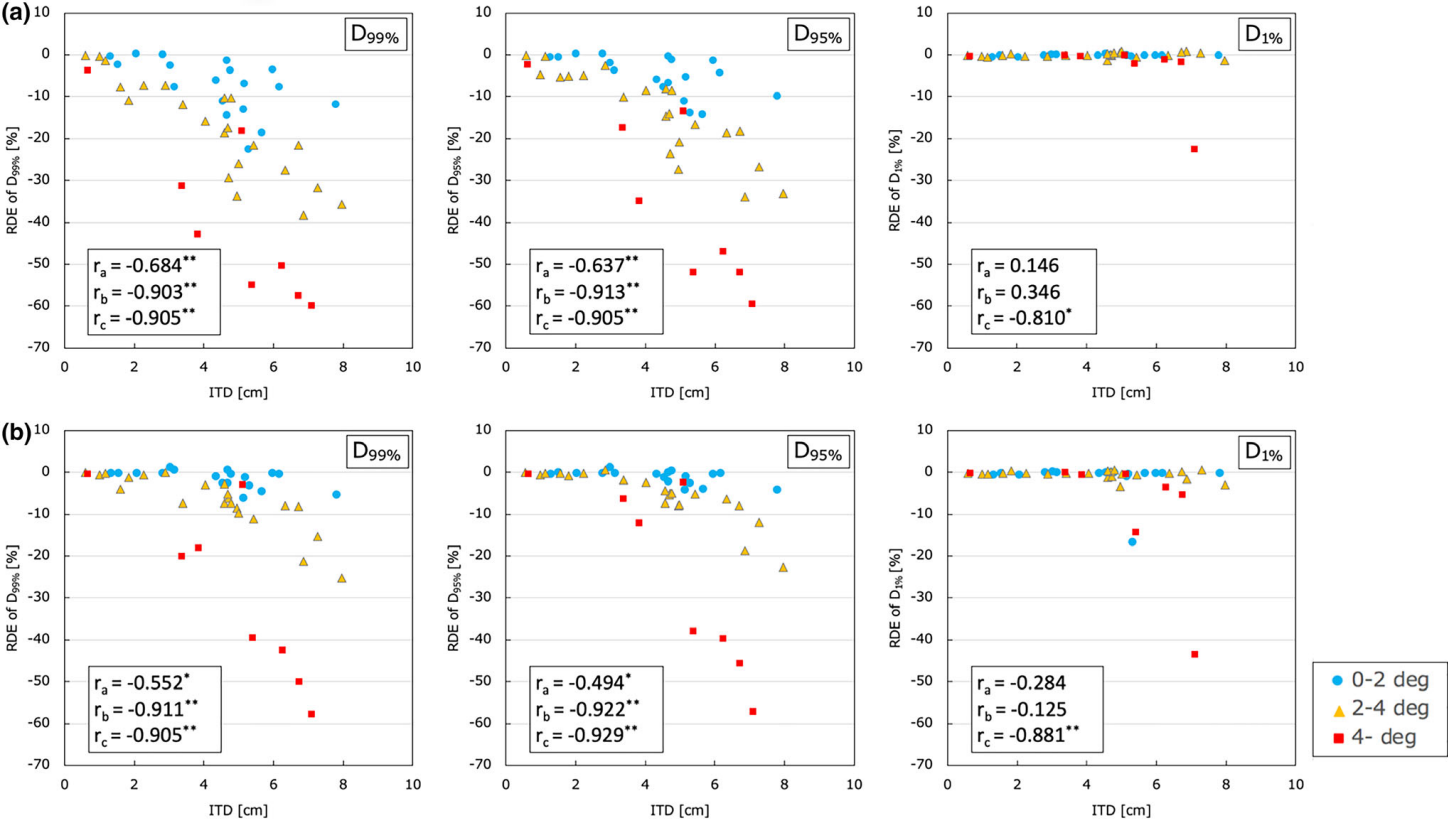
Relative dose error (RDE) of dosimetric parameters for (a) individual PTV and (b) individual GTV, D_99%_, D_95%_, and D_1%_ in ranges of RSE_3D_ (RSE_3D_ ≤ 2°, 2° < RSE_3D_ ≤ 4°, and 4° < RSE_3D_) for multiple‐metastases cases by putting the isocentre‐target distance on the horizon axis. The r_a_, r_b_, and r_c_ are the Spearman's rank correlation coefficients in the ranges of RSE_3D_ ≤ 2°, 2° < RSE_3D_ ≤ 4°, and 4° < RSE_3D_ (*P‐value < 0.05, **P‐value < 0.01).

The ITD and RSE_3D_ were focused on the interaction of dose parameters for individual PTV and GTV values (D_99%_, D_95%_, and D_1%_) of the multiple‐metastases cases (16 patients, 48 targets). In [Fig. 4(a)], for individual PTV, the ITD was found to be correlated with the differences in D_99%_ and D_95%_ in all ranges of RSE_3D_ (RSE_3D_ ≤ 2°, 2° < RSE_3D_ ≤ 4°, and 4° < RSE_3D_). The Spearman's rank correlation coefficients of D_95%_ were −0.64, −0.91, and −0.91 (for RSE_3D_ ≤ 2°, 2° < RSE_3D_ ≤ 4°, and 4° < RSE_3D_, *P* < 0.01). The maximum dose difference of D_95%_ was −59.5%, and the mean was −10.8% ± 14.6%. Regarding the D_1%_, a significant correlation was not observed except for the range of ≥4°. Figure 4(b) for individual GTV shows the similar tendency of correlations between the ITD and the RDE of D_99%_, D_95%_, and D_1%_ in all ranges of RSE_3D_. The dose difference of D_95%_ for GTV was −57.0% at a maximum and −7.0% ± 12.6% on average, which was lower than that for PTV.

**Figure 5:**
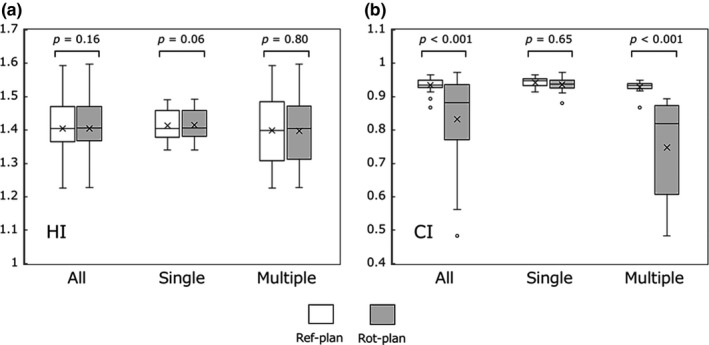
The boxplot of the dosimetric parameters, (a) homogeneity index (HI), and (b) conformity index (CI) for PTV_all_ in the Ref‐plan and Rot‐plan.

Figure 5 shows a boxplot for the (a) HI and (b) CI for PTV_all_. In all cases, the HI was comparable between the Ref‐plan and Rot‐plan (1.41 ± 0.08 vs 1.41 ± 0.08, *P *> 0.05). Although the CI was comparable in the single‐metastasis cases (Ref‐plan vs Rot‐plan, 0.94 ± 0.02 vs 0.94 ± 0.02, *P *> 0.05), the CI was significantly lower in the Rot‐plan than in the Ref‐plan in the multiple‐metastases cases (Ref‐plan vs Rot‐plan, 0.93 ± 0.02 vs 0.75 ± 0.14, *P* < 0.01).

The mean D_max_ for the brainstem was 4.40 ± 4.51 Gy for the Ref‐plan and 4.27 ± 4.31 Gy for the Rot‐plan (*P *> 0.1). Table 1 summarizes the differences between the two treatment plans of dosimetric parameters for normal brain tissues (V_2Gy_ to V_16Gy_ at 2‐Gy intervals). In the single‐metastasis cases, there was no significant difference (*P *> 0.05) for all evaluated parameters. In contrast, in the multiple‐metastases cases, the Rot‐plan provided significantly higher V_10Gy_ (0.08 ± 0.04, *P* = 0.01), V_12Gy_ (0.09 ± 0.07, *P* = 0.02), V_14Gy_ (0.12 ± 0.09, *P* = 0.02), and V_16Gy_ (0.16 ± 0.10, *P* < 0.01) than those in the Ref‐plan.

**Table 1:** Dosimetric parameters for normal brain tissues in the Ref‐plan and Rot‐plan (V_2Gy_–V_16Gy_).

Dosimetric	All (*n* = 29)		Single meta (*n* = 13)		Multiple mets (*n* = 16)	
parameter	difference [cc]	*P*‐value	difference [cc]	*P*‐value	difference [cc]	*P*‐value
V_16Gy_	0.1 ± 0.3	<0.01	0.0 ± 0.0	0.06	0.2 ± 0.3	<0.01
V_14Gy_	0.1 ± 0.2	<0.01	0.0 ± 0.0	0.08	0.1 ± 0.2	0.02
V_12Gy_	0.1 ± 0.1	<0.01	0.0 ± 0.0	0.10	0.1 ± 0.2	0.02
V_10Gy_	0.0 ± 0.1	<0.01	0.0 ± 0.0	0.13	0.1 ± 0.1	0.01
V_8Gy_	0.1 ± 0.1	0.07	0.0 ± 0.0	0.35	0.1 ± 0.1	0.09
V_6Gy_	0.0 ± 0.2	0.36	0.0 ± 0.1	0.65	0.1 ± 0.2	0.35
V_4Gy_	0.1 ± 0.4	0.84	0.0 ± 0.2	0.75	0.1 ± 0.5	0.80
V_2Gy_	−0.1 ± 1.6	0.14	−0.1 ± 0.3	0.55	−0.1 ± 2.0	0.26

## 4 DISCUSSION

In this study, we evaluated the dosimetric effects of clinical rotational setup errors in HyperArc plans for brain metastases. Several studies have reported on patient setup accuracy with thermoplastic mask systems. Gevaert et al. showed that the mean rotational setup errors were −0.09° ± 0.72°, −0.10° ± 1.03°, and 0.23° ± 0.82°, for pitch, yaw, and roll respectively.^26^ Masi et al. reported that the mean rotational setup errors in the three axes were <1.0° for 57 patients for all stereotactic radiotherapy (SRT) fractions.^27^ In this study, the mean rotational setup errors were 0.29° ± 1.23°, 0.00° ± 1.22°, and 0.32° ± 1.27° for pitch, yaw, and roll, respectively, with a maximum of 3.00°, which was comparable with the results of other studies. The use of thermoplastic mask systems allows high accuracy immobilization in LINAC‐based frameless SRS, but this is inferior to that in frame‐based SRS in which the mean error was −0.14° ± 0.25° for pitch, −0.03° ± 0.19° for yaw, and 0.10° ± 0.20° for roll, with a maximum error of 0.87° in flame‐based SRS.^28^ Babic et al*.* reported that rotational setup error was the smallest with one of the frame‐based immobilization devices in six head immobilization devices for both frame‐based and flameless SRS and SRT.^29^ It should be noted that thermoplastic mask immobilization could bring interfractional shifts of ≤3° in patient position.

Roper et al*.* simulated the relationship between rotational setup errors and dosimetric parameters in single‐isocentric VMAT flameless SRS for multitargets cases. Their results showed that D_95%_ values worsened to ≤60% of the prescription dose in uniform rotations of 2° about three axes.^18^ In this study, the HyperArc plans for single‐metastasis cases were robust with respect to rotational setup errors, but the D_99%_ and D_95%_ for each PTV of the Rot‐plan for multiple‐metastases cases decreased considerably. The errors of D_95%_ were slightly bigger than the previous simulation results obtained by Roper. Ohira et al*.* demonstrated that HyperArc plans generated a significantly steeper dose falloff than that of conventional VMAT‐based SRS plans.^14^ Thus, HyperArc plans may be sensitive to rotational setup errors and worsen plan conformity easily. The effect of rotational setup errors on GTV coverage, which was smaller than that on PTV, was also shown. A D_95%_ of GTV located 3 and over cm from isocenter was caused RDE of more than 5% with a high probability in all ranges of RSE_3D_. The effect is dependent on margin size in each institutions and the clinical effect is not clear. Regarding the CI, the CIs for single‐metastases cases under the Ref‐plan and Rot‐plan were comparable with the prescriptions of plans. In contrast, for multiple‐metastases cases, CI significantly decreased depending on the rotational setup errors. According to the report by Garsa et al., the Kaplan–Meier 1‐year local control rate for a CI ≥ 1.75 was 84% compared with the rate for a CI < 1.75 of 69% after controlling for tumor location and volume. They concluded that a lower CI was one of the significant independent predictors of local failure.^30^ Thus, it is important to minimize a decrease in the CI in multiple‐metastases cases. Chang concluded that rotational error could not be ignored for high accuracy and precision treatments, such as SRS, particularly when the distance between the isocenter and target is large.^31^ Gevaert et al. proposed 0.5° as a threshold angle for the correction of rotational setup errors to keep the dose coverage of PTV > 95%.^25^ In recent years, a 6° of freedom treatment couch (6DOF), which can correct rotational setup error based on the 3‐D volumetric image acquired during treatment, is currently available in clinical practice. Gevaert et al. concluded that the 6DOF greatly improved the target positioning with respect to the isocenter clinically.^26^ If the 6DOF was not in use, an additional margin, which compensates for rotational setup error, may be needed for targets located far from the isocenter to avoid underdosage for the target.

Related to the OAR, the Rot‐plan for multiple‐metastases cases caused a significant increase in normal brain tissues in the range of V_10Gy_ to V_16Gy_. Blonigen et al. reported that the V values in the range of V_8Gy_ to V_16Gy_ were the best predictors for the incidence of brain radionecrosis in LINAC‐based SRS.^32^ Thus, the rotational setup errors may induce unexpected radiation side effects. The *D*_max_ for the brainstem for both the Ref‐plan and Rot‐plan were comparable because, in our institution, SRS is not performed for the cases in which the target is located close to critical organs, such as the brainstem. For such cases, fractionated SRT is applied for patients.

Some limitations in this study should be considered. First, the number of patients was limited, and the multiple‐metastases cases were especially not compiled fully. In our institution, SRS is selected after considering the size and location of tumors. Second, this was a retrospective study, so there might have been some bias in the selection of patients. Third, the in‐depth analysis of the effect of the volume of the target on dosimetric parameters for HyperArc plans was not supported. Roper showed that target volume was a strong predictor of D_95%_.^18^ Fourth, the immobilization procedures were not performed by the same therapist, so the quality of immobilization for each patient may not have always been the same. Finally, the intra‐fractional error was not evaluated. HyperArc has been developed to reduce treatment time, so it is important to determine relevance to clinical intra‐fractional motion. Now, there is few reports on rotational intra‐fractional error. Kang et al*.* demonstrated that the maximum intra‐fractional rotational error was ≤ 0.4° with thermoplastic mask during SRS using the CyberKnife.^33^ Despite these limitations, our results provide important information when considering the use of the HyperArc for brain metastases patients and the need for careful assessment of rotational setup errors.

## 5 CONCLUSION

Although the HyperArc plans for the single‐metastasis cases were robust with respect to rotational setup errors, the errors for the multiple brain metastases cases caused statistically significant underdosage for PTV in SRS using the HyperArc. Furthermore, for normal brain tissues, significant increases in the V_10Gy_ to V_16Gy_ values were induced, which are predictors of brain radionecrosis. Consequently, we think that the correction of rotational setup errors is imperative to deliver an adequate dose for multiple‐metastases cases.

## CONFLICT OF INTEREST

The authors are involved in an ongoing collaboration with Varian Medical Systems and financial support was provided.
